# Fish-diversity–inspired multiple soft millirobot system with morphology-encoded selective control

**DOI:** 10.1126/sciadv.aed6170

**Published:** 2026-05-15

**Authors:** Zhengyuan Xin, Zhiqiang Zheng, Yaozhen Hou, Tao Sun, Qing Shi, Toshio Fukuda, Metin Sitti, Huaping Wang

**Affiliations:** ^1^ Key Laboratory of Biomimetic Robots and Systems, Ministry of Education, Beijing Institute of Technology, Beijing 100081, China.; ^2^ Department of Biomedical Engineering, City University of Hong Kong, Hong Kong SAR 999077, China.; ^3^ Physical Intelligence Department, Max Planck Institute for Intelligent Systems, Stuttgart 70569, Germany.; ^4^ Intelligent Robotics Institute, School of Mechatronics Engineering, Beijing Institute of Technology, Beijing 100081, China.; ^5^ Department of Micro-Nano Systems Engineering, Nagoya University, Furo-cho, Chikusa-ku, Nagoya, Aichi 464-8603, Japan.; ^6^ School of Medicine and College of Engineering, Koç University, 34450 Istanbul, Turkey.

## Abstract

Marine ecosystems, particularly coral reef communities, reveal how morphological diversification in fish species facilitates specialized locomotion through evolutionary optimization of body-fin coordination and hydrodynamic adaptations. Inspired by these biomechanical principles, we developed a morphology-encoded patterned magnetic millirobot (MPMR), whose anterior-to-posterior (AP) length ratio and body contour are predefined during fabrication to yield distinct hydrodynamic responses under the same uniform magnetic actuation. These MPMRs, with various morphologies, successfully emulated the undulatory swimming patterns of different fish species in a fluidic environment. Morphological differentiation in MPMRs has been shown to directly influence their motion performance, with an optimal AP ratio (approximately 1:1) and streamlined body contour maximizing propulsion efficiency. Furthermore, MPMRs with distinct morphologies display different frequency-dependent responses to magnetic actuation, leading to morphology-specific velocity profiles. By leveraging these morphology-encoded performance variations, we achieved effective selective control and multitarget delivery of multiple MPMRs under uniform magnetic fields, both in vitro and ex vivo (gastrointestinal tissue). These findings provide a foundation for future designs of flexible millirobots in similar environments and serve as a reference for advancing selective control methods for multiple millirobots in uniform magnetic fields.

## Teaser

Millirobots, inspired by fish diversity, enable selective control in tissues through morphology-encoded differential mobility.

## INTRODUCTION

Propulsion and multirobot coordination are central requirements for untethered millirobots operating in confined, fluid-filled biomedical spaces such as the gastrointestinal tract, where viscous drag, wall contact, and flow disturbances collectively reduce the locomotion efficiency and control precision (*1*–*9*). In such settings, it remains challenging to achieve both efficient undulatory propulsion and selective control of multiple millirobots using a shared, spatially uniform magnetic field.

Marine fish communities, especially coral reef ecosystems, provide a biological analogy to this problem: Morphologically diverse fish can coexist in the same hydrodynamic environment while exhibiting markedly different swimming gaits and performance, owing to evolutionary tuning of body-fin coordination, stiffness distribution, and body contours (*10*–*14*). This observation suggests an opportunity for multimillirobot systems: Rather than relying on environmental heterogeneity or complex field shaping, one may encode morphological diversity into robots so that a uniform actuation signal can elicit differentiated motion responses, thereby enabling selective control.

Prior studies on fishlike robotic swimmers have extensively investigated propulsion mechanisms and the influence of morphology and stiffness on swimming efficiency (*15*–*18*). However, many insights from inertia-dominated regimes do not directly translate to millirobots operating at intermediate Reynolds numbers (*Re*), where viscous effects and fluid-structure interactions (FSIs) can qualitatively alter thrust generation and waveform evolution (*19*, *20*). Recent studies on miniature and soft swimmers have shown that propulsion can be enhanced through parametric optimization of tail architecture (*21*, *22*), stiffness programming (*23*, *24*), and contour refinement (*25*–*27*), including soft robotic platforms that decouple and reprogram undulation patterns (*28*) and stiffness-distribution–driven performance tuning (*29*). However, these studies largely emphasize single-robot speed and efficiency optimization, and they rarely establish a systematic link between morphology-induced hydrodynamic response and selective control of multiple robots under the same uniform magnetic actuation.

Selective or independent control of multiple magnetic robots has been advanced through several strategies. One direction relies on specialized substrates (printed circuit board or microcoil-patterned substrates) or external devices to create localized force fields that either trap robots or program their collective formations (*30*–*33*). Alternatively, selective control can be achieved using specialized boundaries that mechanically anchor individual robots (*34*, *35*). However, relying on specialized substrates or boundaries may reduce versatility for operation in unconfined and deformable biological tissues. A second direction focuses on producing spatially selective magnetic fields or gradients through the modulation of electromagnetic coil control signals or the strategic placement and orientation of permanent magnets. This can steer several robots independently, but it also makes the setup more complicated, and control resolution tends to drop as robots move closer together (*36*–*41*). The third direction takes advantage of the inherent heterogeneity among millirobots to modify their interactions with the surrounding environment, thus producing distinguishable responses under a common input field. This heterogeneity can arise from morphology-dependent hydrodynamic resistance and phase differences (*42*), differences in magnetization orientations that lead to varied wall-contact angles (*43*) and frictional effects, or the use of structurally diverse robots operating in lumen-like network environments (*44*). However, their robustness can be challenged in dynamic luminal environments with variable adhesion and flow.

Here, inspired by fish morphological diversity, we propose a morphology-encoded patterned magnetic millirobot (MPMR) system that leverages morphology-mediated performance differentiation to enable selective control of multiple robots in a uniform oscillating magnetic field. We fabricate alginate-hydrogel MPMRs with a magnetic anterior module and a flexible posterior module, and we systematically tune two key morphological parameters: anterior-to-posterior (AP) length ratio and body contour profile. Combining experiments and FSI simulations, we quantify how morphology shapes undulatory waveform, slip ratio, wake jet angle, and lateral edge velocity, identifying morphologies that maximize propulsion efficiency in intermediate-flow conditions. Crucially, we show that morphological differentiation also produces distinct frequency-dependent response characteristics (including different effective operating ranges and step-out behaviors), which we exploit to realize morphology-specific selective control. Building on this principle, we demonstrate closed-loop selective control and multitarget delivery by multiple MPMRs under uniform magnetic fields in vitro and ex vivo gastrointestinal tissue, establishing a practical route to multirobot selectivity without environmental patterning or spatially varying fields.

## RESULTS

### Bionic and ontology design of MPMRs

**Fig. 1.**
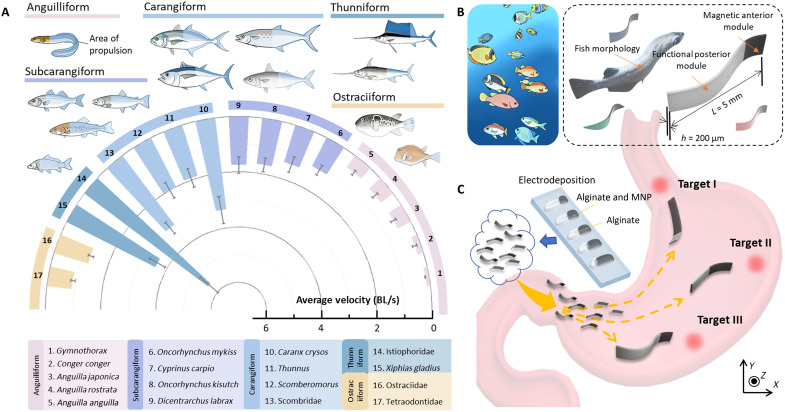
Design of fish-shaped soft magnetic actuated MPMR. (**A**) Modes and average speed of fish (body length per second, BL/s) swimming forward (anguilliform, subcarangiform, carangiform, thunniform, and ostraciiform), classified by body and fin propulsion (indicated by shadow). (**B**) Body design of alginate hydrogel MPMR. Total dimensions of 5 mm (length) by 400 to 600 μm (width) by 200 μm (thickness). (**C**) Schematic illustration of multiple MPMRs in the gastrointestinal tract. Through electrodeposition, drugs, cells, fluorescent particles, and magnetic nanoparticles (MNPs) can be loaded onto various-sized modular regions of MPMRs, enabling the fabrication of MPMRs with diverse morphologies and functionalities. Selective control of multiple MPMRs is achieved by leveraging performance differences arising from morphological differentiation among individual MPMRs. All the images were created by authors. The swimming speed data of various marine fish species were collected from FishBase, an open-access global database of fish species and biological traits (www.fishbase.org/).

Marine fish display exceptional diversity in body morphology and locomotive strategies. As shown in Fig. 1A, interspecies variations in the AP body ratio—defined as the proportion between the rigid anterior region (viscera and skull) and the flexible posterior tail responsible for undulatory motion—are strongly associated with differences in swimming efficiency. Most marine fish rely on body and/or caudal fin (BCF) propulsion, in which thrust is generated by backward-propagating body waves that culminate at the caudal fin. On the basis of characteristic patterns of body deformation and fin involvement, BCF locomotion can be categorized into five classical kinematic modes: anguilliform, subcarangiform, carangiform, thunniform, and ostraciiform (*45*).

Crucially, our analysis reveals that the AP ratio serves as a fundamental biological parameter that regulates the trade-off between undulatory flexibility and hydrodynamic thrust. Specifically, species with higher AP ratios (e.g., anguilliform) rely on large-amplitude body undulations for maneuverability, while those with lower AP ratios (e.g., carangiform) concentrate stiffness anteriorly to maximize thrust transmission efficiency (Fig. 1A). Inspired by this biological mechanism of stiffness distribution, we developed morphology-encoded patterned magnetic millirobots (MPMRs) using electrodeposition technology. Instead of merely replicating the static shape, we abstracted the biological AP ratio into a functional engineering design: a magnetically responsive anterior module (mimicking an actuation-dominant anterior segment that sets body-wave amplitude and phase, while the posterior segment passively propagates the traveling wave) and a soft passive posterior module (mimicking the flexible caudal region). Here, the AP ratio of the MPMR is quantitatively defined as the length ratio of the magnetic anterior module to the nonmagnetic posterior module.

As shown in Fig. 1B, each MPMR comprises these two functionally distinct modules. With total dimensions of 5 mm (length) by 400 to 600 μm (width) by 200 μm (thickness), these millirobots structurally encode the fishlike AP ratios to reproduce the specific wave propagation dynamics identified in our biological analysis. When subjected to an oscillating transverse magnetic field (1 to 10 mT, 1 to 6 Hz), the anterior module undergoes controlled bending, inducing traveling waves along the posterior segment to achieve undulatory propulsion (kinematic model: note S1; actuation system: note S2).

**Fig. 2.**
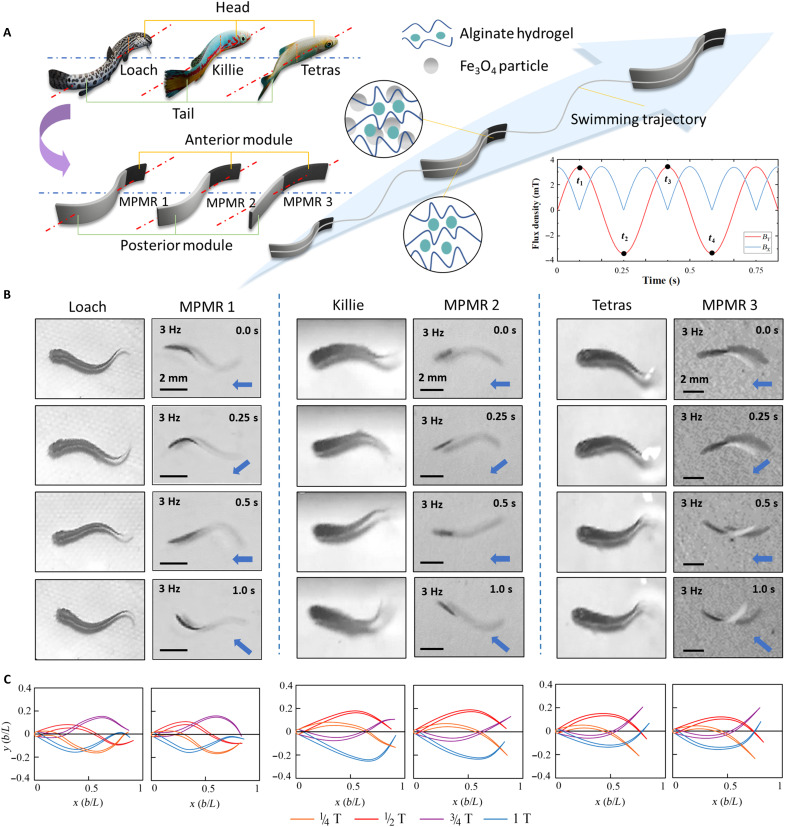
Bioinspired design and posture comparison of MPMRs. (**A**) Morphology of bioinspired MPMRs and magnetic field control. MPMR1, 2, and 3 were established by referring to the morphology of juvenile loach, killifish, and tetras. (**B**) Kinematic comparisons between fish and MPMRs during 1 cycle of near-cyclic swimming (scale bars, 2 mm). The AP ratios of MPMR1, 2 and 3 are respectively approximately the anguilliform, subcarangiform and carangiform in BCF motions. The orientation of the velocity is indicated. (**C**) Comparison of midline kinematic postures between fish and MPMRs. The postures at ^1^/_4_, ^1^/_2_, ^3^/_4_, and 1 T of the motion period were extracted respectively for comparison. The index for evaluating the simulation, namely, the average error of the scanning area, is controlled within 15%.

For instance, three MPMR prototypes (MPMR1 to 3) were designed, mirroring the AP ratios and body contours of juvenile loach, killifish (*Nothobranchius guentheri*), and tetras (*Paracheirodon axelrodi*), as shown in Fig. 2A. Figure 2B illustrates a comparison of the nearly cyclic swimming kinematic postures exhibited by the three types of fish alongside their corresponding bioinspired MPMRs over the course of one complete cycle (movie S1). Quantitative analysis of midline curvature at the postures extracted for ^1^/_4_, ^1^/_2_, ^3^/_4_, and 1 T of the motion period (Fig. 2C) revealed more than 85% waveform similarity between fish and MPMRs, confirming effective biomimetic motion transfer in terms of kinematic generation. However, successfully replicating the waveform does not guarantee optimal propulsion across all morphologies. Variations in the AP ratio lead to notable differences in the motion posture and performance of the three MPMRs. As will be discussed in the following sections, while the bioinspired prototypes function effectively, extreme deviations from optimal morphological ratios result in performance attenuation, thereby validating the morphological constraints observed in nature.

### Effect of AP length ratio on the propulsive efficiency of MPMRs

This section conducted undulatory motion experiments and FSI simulations on MPMRs with different AP ratios under the same magnetic field conditions. The impact of the magnetic anterior module–to–flexible posterior module ratio on the movement of the MPMR in the intermediate Reynolds-number regime was quantitatively evaluated.

**Fig. 3.**
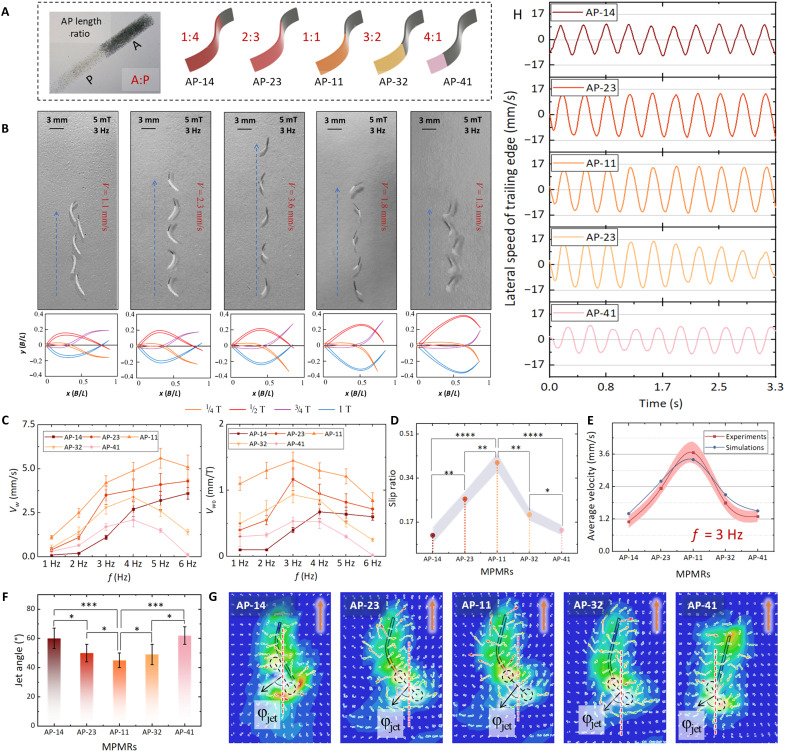
Flow field and kinematic analysis of the impact of the AP length ratio on the propulsive efficiency of MPMRs. (**A**) Designs of soft millirobots (AP-14, AP-23, AP-11, AP-32, and AP-41). (**B**) Experimental study and midline kinematic postures of five MPMRs under a 5-mT, 3-Hz oscillating magnetic field. (scale bars, 3 mm). (**C**) Comparison of the average speed *V_w_* and frequency-normalized swimming speed *V*_*w*0_ variations in the undulatory motion of MPMRs under varying magnetic field frequencies. (**D**) Slip ratio of five MPMRs under a 5-mT, 3-Hz oscillating magnetic field. The findings indicate that the AP-11 exhibits a slip ratio of approximately 0.4, representing an increase of 233% when juxtaposed with the 0.12 slip ratio of the AP-14 [one-way analysis of variance (ANOVA)]. (**E**) A comparison of the average velocity in experimental and simulation studies of undulatory motion in MPMRs. (**F**) Comparison of jet angles in the undulatory motion of MPMRs. The AP-11 reduces jet angle $\varphi_{\text{jet}}$ by 25.2% compared to the AP-14, while the AP-11 achieves an even greater 27.4% reduction relative to the AP-41 baseline (one-way ANOVA). (**G**) FSI phenomena in the flow field simulation of MPMR undulatory motion. (**H**) Lateral velocity variation curve at the tail edge of MPMRs. The AP-11 elevates maximum lateral edge velocity by 105.3% over the AP-14, whereas the AP-41 maintains a substantial 53.2% enhancement in $V_{e}$ compared to the AP-14. Data are presented as mean ± SD (*n* = 5). Statistical analysis: *P < 0.05, **P < 0.01, ***P < 0.001, and ****P < 0.0001.

Five MPMRs [AP-14 (anterior:posterior, 1:4), AP-23, AP-11, AP-32, and AP-41] with a consistent body length of 5 mm were established, featuring magnetic anterior module to nonmagnetic flexible posterior module ratios of 1:4, 2:3, 1:1, 3:2, and 4:1, as shown in Fig. 3A. Five types of MPMRs were placed in a room-temperature liquid environment and subjected to oscillating magnetic fields ranging from 1 to 5 Hz, inducing undulatory motion. The average swimming speed ($V_{w}$) and frequency-normalized swimming speed ($V_{w0}=V_{w}/f$) of the MPMR variations at different frequencies are depicted in Fig. 3C. The velocity deterioration in extreme AP ratios reveals two failure mechanisms: (i) Excessively short posterior modules (AP-41) induce temporally symmetric undulation that compromises low–Reynolds-number propulsion, and (ii) overly long posterior modules (AP-14) suffer from insufficient actuation torque from reduced anterior magnetic domains. Different frequency responses also varied, with MPMRs having shorter magnetic anterior modules and longer posterior modules (AP-14 and AP-23) achieving higher speeds at high frequencies, as shorter anterior modules adapt better to stronger oscillations. In contrast, MPMRs with longer magnetic anterior modules (AP-32 and AP-41) struggled at high frequencies. This performance degradation is attributed to the “step-out” phenomenon inherent in magnetic actuation. As the actuation frequency increases, the hydrodynamic drag and internal structural damping forces grow substantially. When the required driving torque exceeds the maximum magnetic torque available from the anterior module, a substantial phase lag develops between the magnetic field and the robot’s oscillation. This leads to a reduction in the oscillation amplitude of the anterior module, preventing effective wave propagation to the posterior tail and consequently causing a decrease in propulsion speed.

Figure 3B depicts the undulatory motion process of five MPMRs under a 5-mT, 3-Hz oscillating magnetic field (movie S2), along with the changes in their midline kinematic postures over 0.5 cycle of undulatory motion. It is evident that the variation in AP ratios notably influences the undulatory motion postures of the MPMRs, which is the primary factor affecting their propulsion efficiency differences. To further investigate how changes in MPMR motion postures affect kinematics and wake flow structures, we conducted FSI simulations of the undulatory motion for the five MPMRs using Abaqus finite element software. We further calculated the slip ratio ${\bar{\dot{w}}}_{x}/{\bar{v}}_{x}$ to analyze the kinematic differences among the MPMRs. ${\bar{\dot{w}}}_{x}$ represents the average swimming speed, and ${\bar{v}}_{x}$ represents the average propulsive wave speed. The slip ratio can evaluate the energy efficiency of undulatory motion (*46*, *47*). Under identical external conditions, a larger ${\bar{\dot{w}}}_{x}/{\bar{v}}_{x}$ results in higher MPMR motion efficiency. Figure 3D shows the slip ratios during the propulsion of these five MPMRs at a frequency of 3 Hz. The results show that the AP-11 has a slip ratio of approximately 0.4, which is a 233% increase compared to the 0.12 slip ratio of the AP-14.

As shown in Fig. 3E, the comparison between the simulated motion postures of the MPMRs and the experimental results indicates that the simulations accurately replicate the movements of the MPMR. Figure 3E presents the average speeds of the five different AP ratio MPMRs under an oscillation frequency of 3 Hz. The trends in the simulated average speeds closely match the experimental results. This validates the accuracy of the simulation results. The simulations serve as a theoretical tool to screen and identify the optimal structural parameters by revealing hydrodynamic metrics that are difficult to measure experimentally.

Therefore, on the basis of the data obtained from the FSI simulation, this study uses the jet angle $\varphi_{\text{jet}}$ and the lateral edge velocity $V_{e}$ of the MPMR posterior module during motion as specific indicators to quantitatively analyze the kinematic differences among the five MPMRs. These hydrodynamic indicators provide the mechanistic basis for designating the AP-11 configuration as the optimal design choice among the biologically feasible ranges. Figure 3F shows the comparison of the jet angle $\varphi_{\text{jet}}$ for the five MPMRs (*48*). The angle $\varphi_{\text{jet}}$ defines the direction of the shooting flow between two vortices (Fig. 3G). A smaller $\varphi_{\text{jet}}$ indicates that the shooting flow direction is more parallel to the propulsion direction of the MPMR, resulting in less lateral transfer of water momentum and higher swimming efficiency. The lateral edge velocity $V_{e}$ of the MPMR posterior module can be used to evaluate thrust, with previous studies (*19*) indicating that thrust is approximately proportional to ${V_{e}}^{2}$. The comparison of the lateral edge velocity of the MPMRs’ posterior modules is shown in Fig. 3H.

Results indicate that the AP-11 exhibits a 25.2% reduction in jet angle $\varphi_{\text{jet}}$ compared to the AP-14. Relative to the AP-41, the jet angle $\varphi_{\text{jet}}$ is reduced by 27.4%. From the perspective of fluid mechanics, this phenomenon suggests that the AP-11 configuration facilitates the formation of a coherent reverse Kármán vortex street. Compared with extreme aspect ratios (AP-14 or AP-41), the balanced 1:1 proportion optimizes the pressure distribution along the undulating body surface, thereby maximizing the forward momentum component and effectively suppressing lateral energy dissipation. Meanwhile, the maximized $V_{e}$ in AP-11 signifies that the optimized stiffness distribution allows for the most effective accumulation and release of elastic potential energy against the fluid resistance, maximizing thrust production. Therefore, driven by these simulation insights, we confirmed that the AP-11 demonstrates superior propulsion efficiency not merely due to geometric balance but because it maximizes thrust-generating pressure gradients while minimizing wake energy waste. This confirms that the selection of a 1:1 AP ratio is not arbitrary but is the hydrodynamically optimal solution for the combined magnetic-passive actuation method. Although extreme AP ratios (e.g., AP-14 and AP-41) exhibit lower propulsion speeds compared to the optimal 1:1 ratio, this performance differentiation—stemming from intrinsic morphological constraints—is precisely what enables the distinct frequency-response profiles required for the selective control of multiple millirobots demonstrated later.

### Effect of body contour on propulsion efficiency of MPMRs

**Fig. 4.**
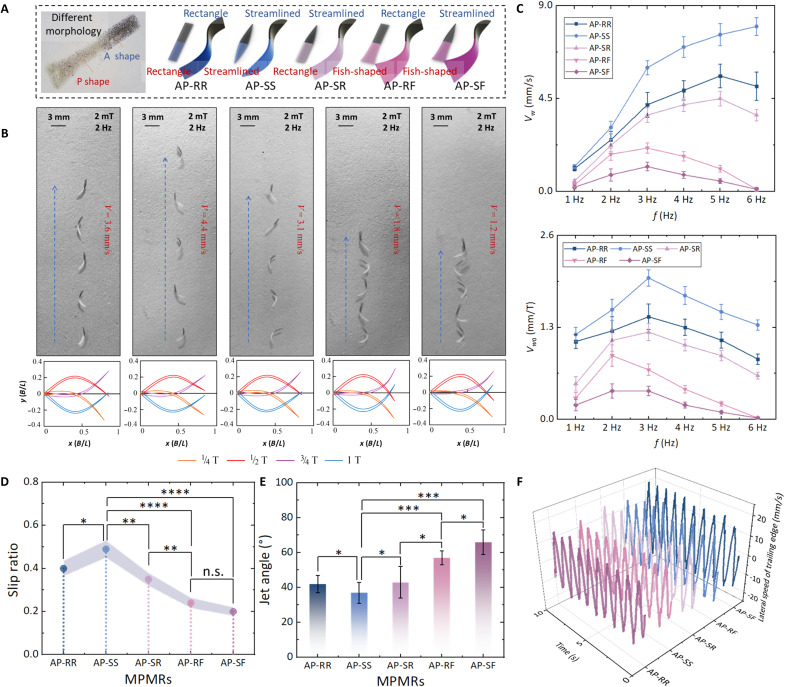
Flow field and kinematic analysis on the undulatory motion of soft MPMRs with different body contours. (**A**) Designs of MPMRs (AP-RR, AP-SS, AP-SR, AP-RF, and AP-SF). (**B**) Experimental study and midline kinematic postures of five MPMRs under a 5-mT, 3-Hz oscillating magnetic field (scale bars, 3 mm). (**C**) Comparison of the average speed *V_w_* and frequency-normalized swimming speed *V*_*w*0_ variations in the undulatory motion of MPMRs under varying magnetic field frequencies. (**D**) Slip ratio of five MPMRs under a 5-mT, 3-Hz oscillating magnetic field. The AP-SS features a slip ratio of 0.49, signifying a 22.5% enhancement in comparison to the AP-RR and an impressive 133.3% increase relative to the AP-SF (one-way ANOVA). (**E**) Comparison of jet angles in the undulatory motion of MPMRs. The results reveal that the AP-SS demonstrates an 11.9% reduction in jet angle compared to the AP-RR. In contrast to the AP-SF, the jet angle is diminished by an impressive 43.9% (one-way ANOVA). (**F**) Lateral velocity variation curve at the tail edge of MPMRs. Compared to the AP-SF, the maximum lateral velocity at the posterior module edge of the AP-SS is elevated by 35.7%. Data are presented as mean ± SD (*n* = 5). Statistical analysis: *P < 0.05, **P < 0.01, ***P < 0.001, and ****P < 0.0001.

Beyond AP ratios, body-contour geometry plays a critical role in determining MPMR propulsion performance. To isolate the effect of contour shape, we examined five MPMRs with a 1:1 AP ratio but distinct contour profiles: AP-RR, AP-SS, AP-SR, AP-RF, and AP-SF. In this notation, R, S, and F correspond to rectangular, streamlined, and fish-tail contours, respectively. For example, AP-SS denotes an MPMR with a 1:1 AP ratio and streamlined anterior and posterior profiles. The average speed $V_{w}$ and frequency-normalized swimming speed $V_{w0}=V_{w}/f$ of the MPMR variations at different frequencies are depicted in Fig. 4C. The streamlined AP-SS exhibits the highest propulsion speed, while AP-RF and AP-SF, with fish tail–shaped designs, show the lowest propulsion speeds. This is because, in the intermediate flow regime, as the Reynolds number decreases, viscous forces gradually replace inertial forces as the primary propulsion forces for MPMRs. The streamlined shape of AP-SS reduces resistance during undulatory motion and allows adaptation to higher oscillation frequencies. In contrast, the fish tail–shaped designs of AP-RF and AP-SF result in greater resistance.

Figure 4B illustrates the undulatory motion of five MPMRs under a 5-mT, 3-Hz oscillating magnetic field, along with the kinematic changes in the midline of the MPMRs during half a cycle of undulatory motion (movie S3). It is evident that the fish tail–shaped design notablely affects the undulatory posture of the MPMRs. The fish tail–shaped posterior modules of AP-RF and AP-SF experience greater lateral resistance, resulting in shorter wavelength undulatory motions and lower average propulsion wave speed (${\bar{v}}_{x}$) at the same frequency. Figure 4D displays the slip ratios (${\bar{\dot{w}}}_{x}/{\bar{v}}_{x}$) during the propulsion of these five MPMRs at a frequency of 3 Hz. AP-SS has a slip ratio of 0.49, representing a 22.5% increase compared to AP-RR and a 133.3% increase compared to AP-SF, which has a slip ratio of 0.21.

We also conducted FSI simulations of the undulatory motion of these five MPMRs at a 3-Hz oscillation frequency. From the simulation results, we extracted the jet angle $\varphi_{\text{jet}}$ and the lateral velocity $V_{e}$ of the posterior module edges for these MPMRs (fig. S4). The comparison of jet angles is shown in Fig. 4E, while the lateral velocity of the posterior module edges is depicted in Fig. 4F.

The fish-tail designs, despite mimicking biological swimmers efficient at high Reynolds numbers, fail at this scale because the abrupt geometric expansion induces larger form drag and unfavorable wake structures. This creates a large low-pressure wake region behind the tail, generating substantial form drag that counteracts the thrust. Conversely, the AP-SS profile optimizes the pressure recovery at the trailing edge. As shown in the jet angle comparison (Fig. 4E) and lateral velocity analysis (Fig. 4F), AP-SS reduces $\varphi_{\text{jet}}$ by 11.9% relative to AP-RR and 43.9% relative to AP-SF while increasing lateral edge velocity by 35.7% compared to AP-SF. This phenomenon indicates that the streamlined shape minimizes the energy lost to generating turbulent structures in the wake. The smooth pressure gradient along the AP-SS body ensures that the energy input from the magnetic field is primarily used to accelerate the fluid axially (thrust). Thus, the streamlined morphology represents an optimal trade-off, maximizing thrust generation while minimizing the energy cost of wake formation in this specific Reynolds-number regime.

The body morphology notably influences the swimming efficiency of MPMRs. In the ocean, many similarly sized fish exhibit vastly different swimming abilities due to variations in body morphology. In this study, although different body morphologies of MPMRs correspond to different optimal propulsion frequencies, MPMRs with a balanced AP ratio and streamlined bodies show a marked improvement in propulsion efficiency. Kinematic analysis reveals that this advantage stems from the optimization of undulatory motion waveforms, smaller jet angles, and greater lateral velocity at the posterior module edge, resulting in greater propulsion force. These MPMRs can be externally controlled via magnetic fields and are promising for applications in liquid-filled cavities within the human body.

### Frequency-dependent velocity differentiation of multiple MPMRs

**Fig. 5.**
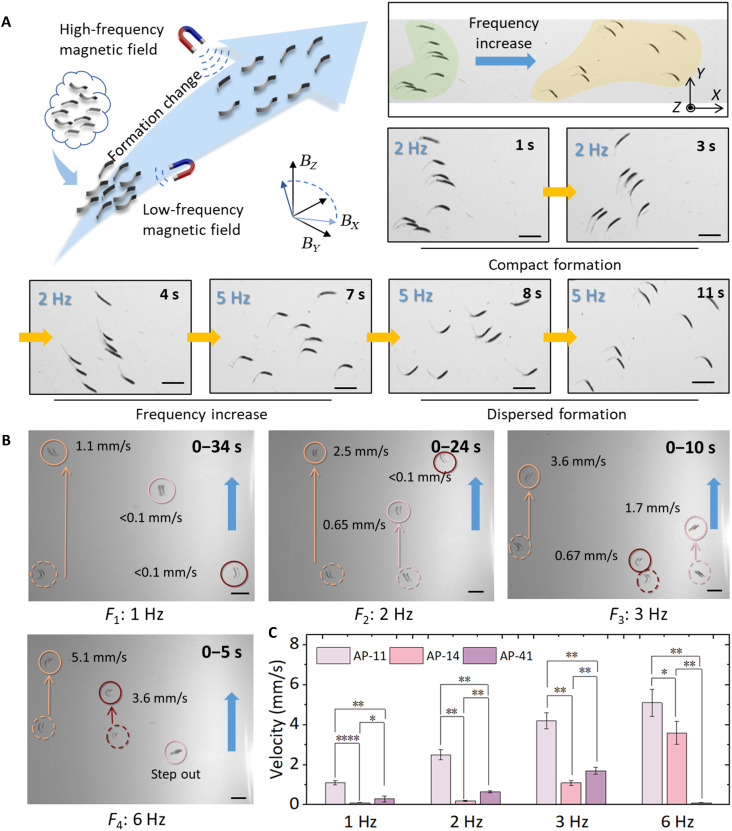
Selective actuation of multiple MPMRs based on body morphological differentiation. (**A**) Demonstration of collective motion of multiple MPMRs. After the magnetic field frequency increases, the aggregated MPMRs gradually disperse (scale bars, 5 mm). (**B**) Experiments on four selective actuation modes for multiple MPMRs (AP-14, AP-11, and AP-41). The three MPMRs exhibited different response performances at different magnetic field frequencies (scale bars, 5 mm). (**C**) Analysis of the significant differences in the movement performance of MPMRs in vitro conditions (two-way ANOVA). Data are presented as mean ± SD (*n* = 5). Statistical analysis: *P < 0.05, **P < 0.01, and ****P < 0.0001.

In Fig. 5A, multiple MPMRs of varying morphologies aggregate and swim together similar to a school of fish. As the magnetic field frequency increases, the differences in their locomotion performance gradually become more pronounced, causing the MPMRs to begin dispersing (movie S4). Morphologically differentiated MPMRs exhibit frequency-dependent locomotion responses under uniform oscillating magnetic fields. Variations in AP length ratios and body contour profiles inherently generate distinct propulsion characteristics through differential FSIs. While this phenomenon manifests physically as a frequency-dependent degradation of performance for nonoptimal morphologies, it creates separable velocity profiles across the population. This differential mobility allows us to prioritize the motion of specific MPMRs under identical field conditions. Consequently, effective selective control can be achieved by leveraging these relative velocity differences in combination with time-division multiplexing, thereby eliminating the need for environmental heterogeneity. The selective control of multiple magnetically actuated MPMRs, achieved through real-time adjustments of magnetic field parameters, allows for rapid switching of control targets (such as specific individuals or subgroups) to adapt to the dynamic task requirements in complex environments.

The study placed AP-14, AP-11, and AP-41 (rectangular MPMRs with AP ratios of 1:4, 1:1, and 4:1, respectively) simultaneously in environments with varying magnetic field frequencies. The average speeds of these three MPMRs at different frequencies are shown in Fig. 3C. AP-14 exhibits an effective operating frequency range of 3 to 6 Hz, AP-11 ranges from 1 to 6 Hz, and AP-41 operates effectively between 2 and 5 Hz. Consequently, four control modes were established at frequencies of 1, 2, 3, and 6 Hz (Fig. 5B and movie S5). At 1 Hz, the magnetic field independently actuates AP-14 at a speed of 1.1 mm/s. At 2 Hz, it selectively drives AP-11 and AP-41 at speeds of 2.5 and 0.65 mm/s, respectively. At 6 Hz, AP-14 and AP-11 are actuated with speeds of 3.6 and 5.1 mm/s, respectively. At 3 Hz, all three MPMRs (AP-14, AP-11, and AP-41) are driven simultaneously at speeds of 0.67, 3.6, and 1.7 mm/s, respectively. The performance variations induced by morphological differentiation were statistically validated through locomotion comparisons, demonstrating significant velocity differences among distinct morphological designs under identical actuation parameters (Fig. 5C and note S3).

### Selective control strategy of multiple MPMRs for multi-target navigation and delivery

**Fig. 6.**
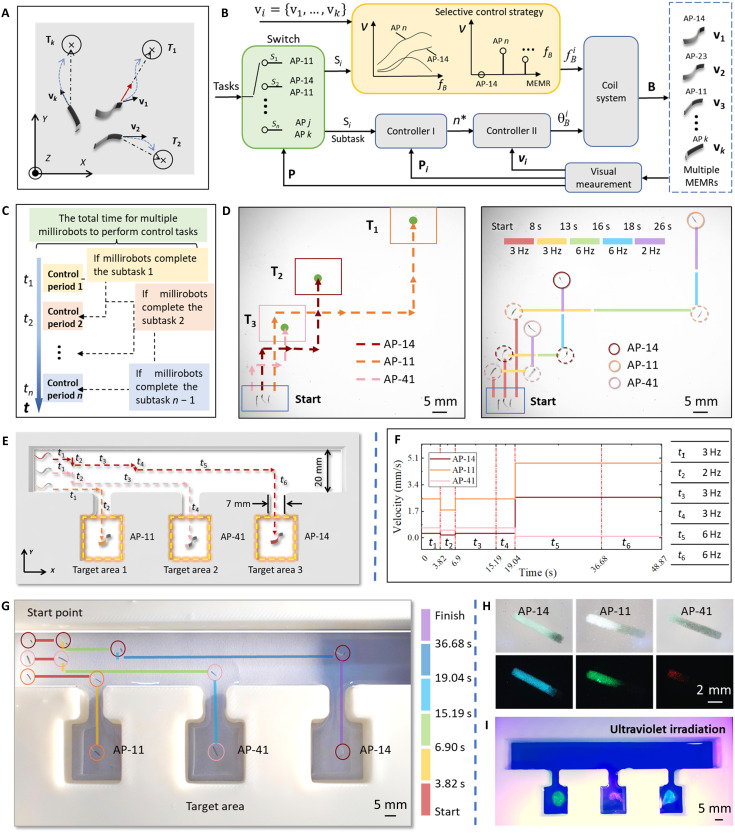
Selective control strategy of multiple MPMRs. (**A**) Tasks of multiple MPMRs for selective control. (**B**) Block diagram for selective control strategy of multiple MPMRs. The task assigned to multiple MPMRs for autonomous positioning control or trajectory following is decomposed into discrete subtasks, which are executed sequentially by these MPMRs. (**C**) Schematic of time-division multiplexing. (**D**) Selective control of three MPMRs initiated from a common origin and guided to distinct target areas under a general experimental setup (scale bars, 5 mm). (**E**) Schematic diagram of multiple MPMR selective operation experiments (AP-14, AP-11, and AP-41). (**F**) Control strategies for MPMRs based on selective control. The variations in velocity corresponding to different time instances and frequencies of the three MPMRs are presented. (**G**) Collective operation of multiple MPMRs under a uniform field. Three MPMRs start from the same origin, each reaching different target areas to release their carried fluorescent particles (scale bar, 5 mm). (**H**) MPMRs carrying fluorescent particles of different colors. AP-14, AP-11, and AP-41 are equipped with blue, green, and red fluorescent particles, respectively (scale bar, 2 mm). (**I**) Millirobots successfully delivering fluorescent particles (scale bar, 5 mm).

On the basis of these selective actuation modes, control of multiple MPMRs to different target positions and along distinct reference paths was achieved, as shown in Fig. 6A. To achieve precise multiple MPMR coordination in complex environments, we established a hierarchical closed-loop control framework depicted in Fig. 6B, which integrates visual feedback, time-division multiplexing, and dual-stage controller architecture. The green block encloses the switch of time-division multiplexing, and the yellow block encloses the selective actuation strategy. The overall system operates by autonomously guiding *n* subgroups of MPMRs from initial positions **P** to goal position $G=\left\{G_{1},\ldots ,G_{n}\right\}$ along distinct reference paths set by a global planner.

The control strategy relies on a time-division multiplexing method that discretizes the global task into short, sequential subtasks (Fig. 6C). In this scheme, the system switches control authority to the *i*th subgroup *S_i_* during the *i*th control cycle based on visual position feedback; the cycle transitions only when the current active subgroup reaches its designated subgoal, ensuring conflict-free actuation sequences. In the *i*th control cycle, the *i*th MPMR subgroup $S_{i}=\left\{{\text{AP}}_{1},\ldots ,{\text{AP}}_{k}\right\}$ with current positions $P_{i}=\left\{p_{1},\ldots ,p_{k}\right\}$ and velocities $v_{i}=\left\{v_{1},\ldots ,v_{k}\right\}$ is actuated and steered toward the desired goal positions $G_{i}=\left\{g_{1},\ldots ,g_{k}\right\}$ following the reference paths.

The core control logic is implemented through two cascaded controllers designed to translate path deviations into magnetic actuation signals. Given that the MPMRs in a subgroup are actuated by a uniform global magnetic field, the controllers optimize the collective performance. Controller I (path following) functions as a high-level planner that determines the optimal collective desired direction $n^{*}\left(n_{x}^{*},n_{y}^{*}\right)$ for the active subgroup *S_i_*

$$n^{*}=\sum_{j=1}^{k}w_{j}\left(\frac{M}{\left\|d_{j}\right\|+M}r_{∥,j}+\frac{\left\|d_{j}\right\|}{\left\|d_{j}\right\|+M}r_{⊥,j}\right)$$ (1)

As defined in Eq. 1, this controller calculates a weighted vector sum of two components: the path-tangential vector $r_{∥}$ (guiding forward motion) and the path-normal vector $r_{⊥}$ (correcting cross-track error **d***_j_*). Intuitively, the factor *M* serves as a tunable gain that balances the convergence speed against path adherence; a smaller *M* prioritizes rapid convergence to the path, while a larger *M* ensures smoother motion along the path tangent. *w_j_* is the task-dependent weight of the MPMRs in the active subgroup *S_i_*, $\sum_{j=1}^{k}w_{j}=1$. Controller II (heading control) acts as the low-level execution layer, minimizing the weighted aggregate angular error $\theta_{e}$ between the desired collective direction $n^{*}\left(n_{x}^{*},n_{y}^{*}\right)$ and the actual velocity of the robots $v_{j}=\left(v_{j,x},v_{j,y}\right)$ using a proportional-integral algorithm

θet=∑j=1kθe,j=∑j=1kwj[atan2(ny∗,nx∗)−atan2(vj,y∗,vj,x∗)] (2)

where $\theta_{e}$ represents the weighted deviation of the subgroup’s velocity vectors from the desired direction $n^{*}$. Consequently, the orientation angle θBt of the uniform magnetic field applied to subgroup *S_i_* at time step *t* is updated as

θBt=θBt−1+kpθet+ki∑θet (3)

where *k*_p_ and *k*_i_ are the proportional and integral coefficients, respectively. When the MPMR subgroup *S_i_* reaches the target positions, it executes the next control subtask, with the sequence determined by time-division multiplexing switches.

In the actual system implementation, the proportional coefficient *k*_p_ is tuned first to ensure rapid response to direction changes, followed by the integral coefficient *k*_i_, which is adjusted to eliminate steady-state directional errors caused by fluid resistance or magnetic field nonuniformities.

The core mechanism of our selective control lies in the frequency-dependent velocity differentiation rather than simple binary activation. As indicated in Fig. 5 (B and C), different morphologies (e.g., AP-11 versus AP-41) exhibit distinct velocity profiles across the frequency spectrum. Unlike sequential control that relies solely on stopping one robot to move another, our strategy exploits the relative velocity differences induced by morphology. To demonstrate the robustness of this strategy, we conducted experiments in both a general open space and a complex terrain with obstacles. In the general scenario (Fig. 6D and movie S6), to verify the multitarget tracking capability, three MPMRs (AP-14, AP-11, and AP-41) were initially positioned at the same starting point. By modulating the actuation frequency, we achieved asynchronous trajectory tracking to guide them to three distinct spatial targets.

Furthermore, to showcase adaptability in structured environments, we created a terrain with three distinct target areas. Starting from the same origin, three MPMRs were guided using different modes of selective control to reach their respective target areas and release the carried fluorescent particles, as shown in Fig. 6E. Figure 6F illustrates the control scheme for the collaborative control of the multiple MPMRs. As illustrated in Fig. 6G, during the *t*_1_ phase, under the influence of a 3-Hz oscillating magnetic field, AP-11 swiftly accelerates to be the first to arrive at the entrance of target area 1. In the subsequent *t*_2_ phase, operating within a 2-Hz magnetic field, both AP-11 and AP-41 progress downstream, with AP-11 successfully entering target area 1. During the *t*_3_ and *t*_4_ phases, once again under a 3-Hz magnetic field, AP-41 attains target area 2 at an enhanced velocity. Ultimately, during the *t*_5_ and *t*_6_ phases, under a 6-Hz magnetic field, AP-41 further amplifies its speed to reach target area 3 (movie S7). After the MPMRs reach their respective target areas, a sodium citrate solution is injected into the liquid environment, causing the calcium alginate bodies of the MPMRs to dissolve and release different colored fluorescent particles they carry, completing the targeted delivery, as illustrated in Fig. 6 (H and I).

Quantitative characterization of the dissolution-triggered payload release was conducted by fluorescence image analysis. Because the payload (fluorescent particles) was initially immobilized inside the Ca-alginate hydrogel, release occurred progressively, as the MPMR body dissolved and the particles dispersed from an aggregated state to the surrounding fluid. We quantified the release efficiency as the fraction of fluorescence intensity transferred from the robot body to the surrounding region of interest (ROI), $\eta \left(t\right)=I_{\text{out}}\left(t\right)/\left[I_{\text{in}}\left(t\right)+I_{\text{out}}\left(t\right)\right]$, where $I_{\text{in}}$ and $I_{\text{out}}$ denote the integrated fluorescence intensity inside and outside the robot contour, respectively. Under the citrate triggering condition, the MPMRs exhibited a rapid increase of η(t) and reached $\eta_{\text{final}}=94.2\pm 5.8\%$ within 260 s (*n* = 6), as shown in fig. S7.

We further evaluated release positioning accuracy by measuring the distance between the target area center and (i) the MPMR centroid at the onset of release and (ii) the fluorescence centroid at *t*_90_ [the time when η(t) first reaches 90% of its final value]. The mean onset error *e*_start_ and *t*_90_ deposition error *e*_90_ were *e*_start_ = 3.2 ± 1.3 mm and *e*_90_ = 0.85 ± 0.12 mm (*n* = 5), respectively. These quantitative results support the feasibility of MPMR-enabled localized delivery with controllable release timing and spatial accuracy.

### Ex vivo manipulation of MPMRs

**Fig. 7.**
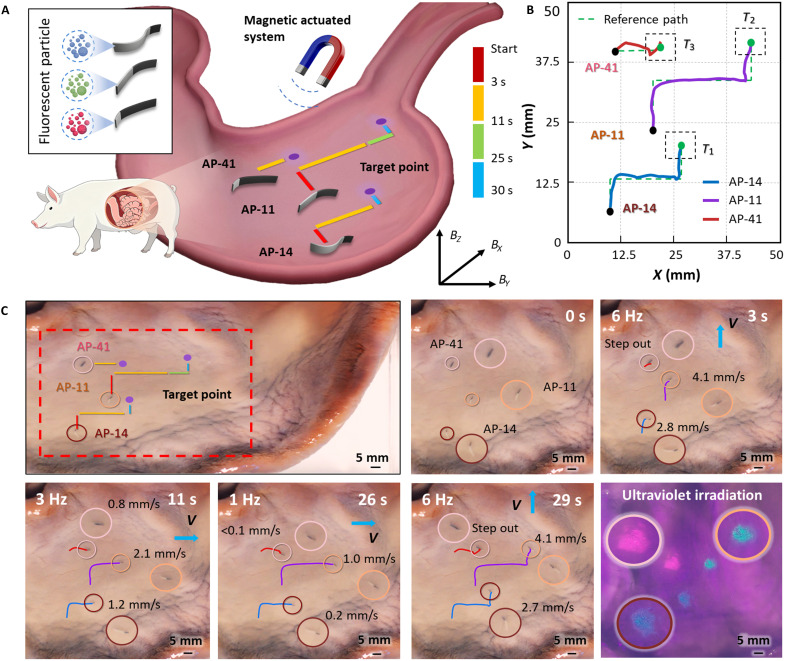
Ex vivo manipulation of multiple soft MPMRs based on selective control. (**A**) Schematics of the experiment. Through selective control of multiple MPMRs in an ex vivo porcine stomach, three randomly placed MPMRs (AP-14, AP-11, and AP-41) were driven to reach three different random zones and successfully release fluorescent particles. (**B**) Trajectory of the MPMRs in the ex vivo porcine stomach. *T* is the subtask completed by the corresponding MPMR. (**C**) The MPMRs performed selective control movements in the ex vivo porcine stomach and successfully released fluorescent particles. AP-14, AP-11, and AP-41 are equipped with blue, green, and red fluorescent particles, respectively (scale bars, 5 mm). All ex vivo porcine stomachs are purchased from Taobao, China.

To evaluate whether multiple MPMRs can reliably move and be selectively controlled in realistic biological settings, we carried out ex vivo tests in gastrointestinal tissues. We first used porcine gastric tissue to validate basic locomotion and multimode selectivity (Fig. 7A). Three morphologically distinct MPMRs (AP-14, AP-11, and AP-41), each loaded with a different color of fluorescent particles, were randomly dispersed within the tissue. Trajectory tracking showed that all three robots preserved stable directional motion across the four selective control modes despite the confined, adhesive environment (Fig. 7B). After navigation, local injection of sodium citrate dissolved the Ca-alginate hydrogel within 5 min, releasing the fluorescent particles at the preset targets (Fig. 7C and movie S8).

**Fig. 8.**
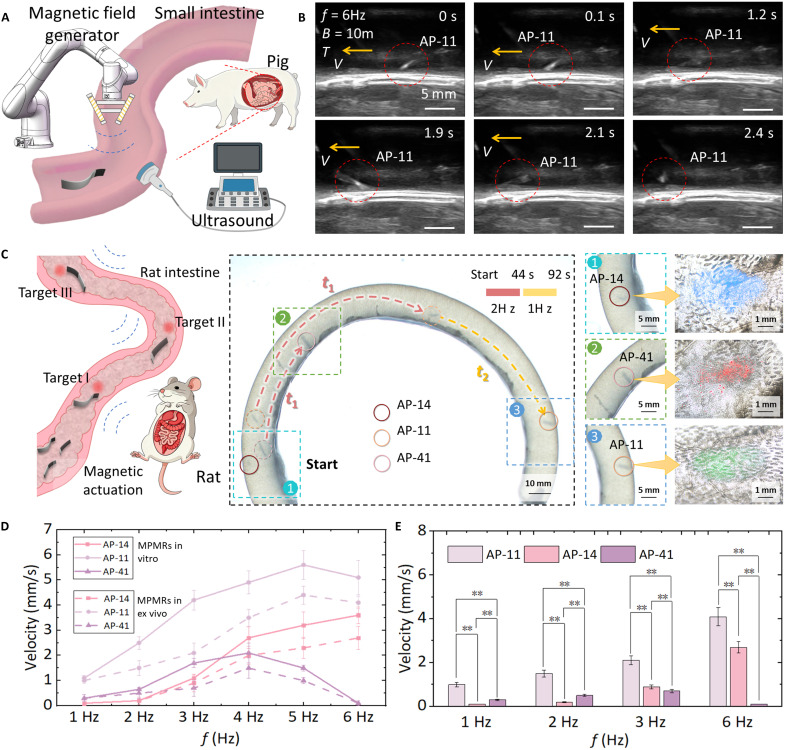
Multirobot selective control and multitarget delivery in intestinal tissues. (**A**) Schematic diagram of the movement of MPMRs in the ex vivo porcine small intestine under medical imaging. The platform included a magnetic control system (a three-stage magnetic control coil on a six-axis robotic arm) and an imaging system (ultrasound). (**B**) Ultrasound images of MPMRs moving in the ex vivo porcine small intestine. (**C**) Multirobot selective control and multitarget delivery in an ex vivo rat intestine. (**D**) Comparison of the motion performance of MPMRs in vitro fluidic environments and intestinal tissues. (**E**) Analysis of the significant differences in the movement performance of MPMRs in intestinal tissues (two-way ANOVA). Data are presented as mean ± SD (*n* = 5). Statistical analysis: **P < 0.01. All ex vivo porcine small intestines are purchased from Taobao (China). Sprague-Dawley rats were purchased from the Peking University Laboratory Animal Center (Beijing, China).

We then examined the operational feasibility of morphology-encoded selective control in an intestinal lumen under clinically relevant imaging. Ex vivo porcine small-intestine experiments were performed under ultrasound guidance using an integrated platform consisting of (*1*) morphology-encoded MPMRs, (*2*) a clinical ultrasound system, and (*3*) a six-df robotic arm with triaxial electromagnetic actuation (Fig. 8A). Real-time ultrasound confirmed successful navigation through the lumen (Fig. 8B, fig. S5, and movie S9).

To directly demonstrate why selective control matters—and that it is achievable in a lumen—we designed a multitarget delivery task in real intestinal tissue. Three morphologically distinct MPMRs (AP-14, AP-11, and AP-41), carrying blue, green, and red fluorescent particles, were introduced into an ex vivo rat intestinal segment (inner diameter ~ 10 mm). Using the same spatially uniform oscillating magnetic field, the robots were selectively guided to three predefined target zones via the proposed frequency-encoded actuation combined with time-division multiplexing. Once each robot reached its assigned site, localized sodium citrate injection triggered rapid dissolution and released the color-coded payloads at the intended locations (Fig. 8C and movie S10).

Last, we quantified how the biological environment affects locomotion. Compared with in vitro hydrodynamic conditions, propulsion velocities in the lumen decreased by ~30 to 50% (e.g., AP-11 dropped from 4.2 to 2.1 mm/s at 3 Hz), mainly due to mucosal adhesion and resistance from tissue surface (Fig. 8D). Morphology-dependent performance differences persisted in the lumen and were statistically significant under identical actuation parameters (Fig. 8E and note S3). Together, these results confirm that morphology-encoded selective control remains robust in complex ex vivo tissues. In addition, we investigated the effects of different fluid viscosities and background flow patterns on the locomotion performance of MPMRs. These findings further confirm the feasibility of their future application in the complex and dynamic environment of the gastrointestinal tract in vivo (note S4).

## DISCUSSION

We developed an MPMR and established a morphology-mediated design paradigm for soft MPMRs operating in intermediate Reynolds-number regimes, where bioinspired morphological variations fundamentally govern both propulsion efficiency and selective control capability of multiple MPMRs. Our modular fabrication approach enables systematic tuning of two critical morphological parameters: AP length ratios and body contour profiles.

By altering the MPMR morphology and using magnetic field control, different swimming patterns are simulated. The analysis focuses on factors such as slip ratio, motion waveform, jet angle, and the edge velocity of the posterior modules, integrating experimental data with FSI simulations to elucidate efficiency changes. Results indicate that, at the same frequency of 3 Hz, MPMRs with the AP ratio of 1:1 exhibited a 232% increase in average speed compared to those with a ratio of 1:4. Under identical conditions, streamlined MPMRs (AP-SS) outperformed rectangular MPMRs (AP-RR) by 11.9% in speed. Kinematic analysis suggests that this improvement is due to enhanced wave characteristics in the 1:1 ratio and streamlined body design, leading to better energy utilization and thrust efficiency.

This morphological differentiation principle enables frequency-selective control of multiple MPMRs in uniform magnetic fields. Our experimental validation shows that geometrically distinct MPMRs exhibit differential frequency locking behaviors (3- to 6-Hz range), allowing sequential activation through field frequency modulation. In both vitro and ex vivo gastrointestinal tissue experiments, selective control of multiple MPMRs was achieved, with different MPMR reaching distinct target points and releasing fluorescent particles of varying colors under selective magnetic control. Our advances are orthogonal to, yet build upon, prior fishlike soft swimmer studies that focus on undulatory propulsion mechanisms and single-robot performance tuning via stiffness distribution or body design 0 (*28*, *29*). Unlike these works, we explicitly exploit intermediate-*Re* morphology-frequency coupling to obtain separable frequency-response curves across a population, thereby enabling distinguishable frequency-response curves across a population. By mapping these performance differentiations, we realize the selective control of multiple robots under one shared, spatially uniform oscillating magnetic field. This positioning also distinguishes our approach from swarm demonstrations that depend on spatially nonuniform fields to drive aggregation and separation and are often geared toward single-target delivery (*49*). At the same time, it complements independent-control strategies that differentiate robots through magnetization- or contact-mediated effects such as contact-angle and friction contrasts (*42*, *43*). Because contact and local environmental conditions can vary widely in soft, adhesive lumens, we instead emphasize morphology-mediated differences in magnetic field response as an intrinsic “encoding” to reduce the sensitivity of control strategies to environmental variability. We show that the resulting response separation persists in ex vivo gastrointestinal tissue, supporting the feasibility of morphology-encoded selectivity in clinically relevant, variable environments.

However, although our MPMRs are inspired by the diversity of fish, fundamental differences persist: Fish locomotion leverages active neuromuscular control and conformal fin dynamics across multiple body segments for efficiency, whereas MPMR motion relies on the passive propagation of magnetically induced traveling waves from a single anterior module in viscosity-dominated regimes, emphasizing drag minimization over biologically analogous thrust generation mechanisms. While morphological differentiation enables selective control under uniform fields, the achievable resolution and precision are inherently linked to the frequency-dependent response characteristics and currently lag behind the fine neuromuscular control exhibited by actual fish. Last, while validated ex vivo, broader in vivo testing across diverse anatomical sites is required to fully assess the translational potential and interaction dynamics within living systems.

## MATERIALS AND METHODS

### MPMR fabrication process

A deposition solution for alginate hydrogel was prepared by mixing 1% (w/v) sodium alginate and 0.25 to 0.75% (w/v) calcium carbonate. Fe_3_O_4_ powder was added to the deposition solution to form the magnetic anterior module of the MPMR, while the solution without magnetic powder was used to create the flexible posterior module. The deposition solution containing magnetic powder and the solution without it were dripped onto anodic plates with different patterns in varying ratios. A cathodic plate was then placed over the solutions, as shown in fig. S6. By adjusting the ratio of the two solutions and the patterns on the anodic plates, MPMRs with varying AP ratios and body contours can be fabricated. A 3 to 5-V dc voltage was applied between the fluorine-doped tin oxide layers of the anode and cathode plates for 5 to 10 s, inducing an electrodeposition reaction that formed the calcium alginate hydrogel MPMR. The anode plate with the MPMR was then placed in a 1-T directional magnetic field for magnetization. Last, the anode plate was rinsed to separate the calcium alginate structure, yielding the MPMR.

Within the internal structure of the MPMR, the functional acidic groups (e.g., −COO^−^) interact with monovalent cations (such as Na^+^ in sodium citrate). These interactions promote water uptake and lead to the formation of a loose gel network, thereby facilitating the release of encapsulated drugs or cells for targeted delivery.

### The range of *Re* for the MPMR at room temperature

The Reynolds number (*Re*) is a dimensionless quantity used to characterize fluid flow, helping to determine the resistance experienced by an object moving through a fluid. A smaller Reynolds number indicates greater influence of viscous forces, while a larger number indicates greater influence of inertial forces. All quantitative studies were conducted at room temperature, 23° ± 1.5°C, with an average kinematic viscosity of $9.3\times 10^{-7}$ m^2^/s. In our current research, the speed of the MPMRs ranges from 1.0 to 8.1 mm/s. The dimensionless *Re* is given by Eq. 4

$$\text{Re}=\frac{\rho_{w}{\bar{\dot{w}}}_{x}l_{r}}{\mu_{w}}=\frac{{\bar{\dot{w}}}_{x}l_{r}}{n_{w}}$$ (4)

where $\rho_{w}$ is the density of water, ${\bar{\dot{w}}}_{x}$ is the average swimming speed along the propulsion direction, *l_r_* is the body length (5 mm), $\mu_{w}$ is the dynamic viscosity, and $n_{w}$ is the kinematic viscosity. Calculations reveal that the *Re* range for the MPMR’s swimming environment is between 5.37 and 43.5, indicating that it operates within the intermediate flow regime.

### FSI simulation design

This study uses Abaqus/CAE 2018 to establish a three-dimensional transient FSI model, systematically analyzing the dynamic response of heterogeneous MPMRs in an intermediate Reynolds-number flow regime. The simulation model consists of two coupled domains: (i) a cylindrical fluid domain with a diameter of 8 mm and a length of 30 mm, using a structured hexahedral mesh (minimum element size of 0.1 mm), with properties set for the aqueous solution (kinematic viscosity μ = 9.3 × 10^−7^ m^2^/s, density ρ = 1000 kg/m^3^); (ii) the flexible body domain of the microrobot, modeled using a hyperelastic constitutive model (parameters: elastic modulus μ_1_ = 500 Pa, Poisson’s ratio = 0.5). Bidirectional coupling between fluid and structure is achieved through the arbitrary Lagrangian-Eulerian adaptive meshing technique, with the solver using an explicit dynamics module (time step Δ*t* = 10 s). Experiments are conducted under uniform boundary conditions (initial velocity of 0 m/s, head oscillation frequency of 3 Hz).

### Ex vivo tests in rat intestines

The intestines were obtained from Sprague-Dawley rats received from the Peking University Laboratory Animal Center (Beijing, China). The animals were housed under room temperature of 20° ± 1°C, relative humidity of 50 ± 10%, and 12-hour light/12-hour dark cycle and received food and water ad libitum. The rats were euthanized, the abdominal cavity was opened, and the intestines were removed from the stomach. The MPMRs was injected into the intestine that was filled with artificial intestinal liquid (Solarbio, Beijing, China), and the ends of the intestine were then sealed using two plastic clips. The MPMRs was manipulated using a magnetic system.

### Definition of the jet angle

In the context of the FSI analysis presented for the MPMRs, the “jet angle” ($\varphi_{\text{jet}}$) serves as a quantitative metric describing the hydrodynamic orientation of the momentum-carrying fluid jet generated between shedding vortices in the wake. Specifically, as illustrated in Fig. 3G and rooted in the mechanics of undulatory propulsion where a vortex street is typically induced, $\varphi_{\text{jet}}$ is defined as the angle subtended between the directional vector of the “shooting flow”—the concentrated fluid jet induced between two consecutive vortices—and the longitudinal axis of the MPMR’s propulsion direction (fig. S3).

Physically, as the flexible posterior module undergoes undulation, it generates a wake structure where the fluid momentum flux, or shooting flow, emerges between the shed vortices; therefore, this angle serves as a critical metric for propulsion efficiency, where a minimized $\varphi_{\text{jet}}$ indicates that the hydrodynamic jet is aligned more parallel to the swimming direction, thereby reducing lateral momentum dissipation and maximizing the effective forward thrust derived from the vortex wake.

### Quantification of payload release and positioning accuracy

Quantification of payload release was performed using ImageJ. The MPMR contour was segmented from bright-field images (or fluorescence thresholding before dissolution) and used to compute the integrated fluorescence intensity inside the robot body (*I*_in_). A fixed ROI covering the target area and its vicinity was used to compute the fluorescence intensity outside the robot contour (*I*_out_). Release efficiency was then defined as the fraction of fluorescence located outside the robot body

$$\eta \left(t\right)=\frac{I_{\text{out}}\left(t\right)}{\left[I_{\text{in}}\left(t\right)+I_{\text{out}}\left(t\right)\right]}$$ (5)

From the resulting η(t) curves, characteristic release times were extracted: *t*_50_, and *t*_90_, corresponding to the time points at which η(t) first reached 50 and 90% of the final release level, respectively.

Release positioning accuracy was quantified by the Euclidean distance between the target center and the MPMR centroid at release onset (*e*_start_) and between the target center and the fluorescence intensity centroid at t_90_ (*e*_90_). All metrics are reported as mean ± SD with *n* = 5 independent trials.

## Supplementary Materials

### The PDF file includes:

Supplementary Text Notes S1 to S4 Figs. S1 to S8 Tables S1 and S2 Legends for movies S1 to S10
