# Supplementary material for: Fish-diversity–inspired multiple soft millirobot system with morphology-encoded selective control
Source: Sci Adv. 2026 May 15;12(20):eaed6170. doi: 10.1126/sciadv.aed6170 (PMC13178541; doi:10.1126/sciadv.aed6170)
Supplement: Supplementary file 1 — Supplementary Text Notes S1 to S4 Figs. S1 to S8 Tables S1 and S2 Legends for movies S1 to S10 [file sciadv.aed6170_sm.pdf]

Science Advances
AAAS

Supplementary Materials for

Fish-diversity–inspired multiple soft millirobot system with morphology-encoded selective control

Zhengyuan Xin *et al.*

Corresponding author: Metin Sitti, sitti@is.mpg.de; Huaping Wang, wanghuaping@bit.edu.cn

Sci. Adv. **12**, eaed6170 (2026)
DOI: 10.1126/sciadv.aed6170

The PDF file includes:

Supplementary Text
Notes S1 to S4
Figs. S1 to S8
Tables S1 and S2
Legends for movies S1 to S10

Other Supplementary Material for this manuscript includes the following:

Movies S1 to S10

Supplementary Text

Note S1. Kinematic Model for Undulatory Motion of MPMRs

A schematic diagram of the undulatory motion in MPMRs is shown in Fig. S1. Let the oscillating magnetic field with a frequency of f and a magnetic field strength of B be denoted as $\mathbf{B}_w$. The equation of undulatory swimming motion for the MPMR, derived under the force-free swimming condition, is expressed as:

$$\begin{pmatrix} \mathbf{U} \\ \mathbf{\Omega} \end{pmatrix} = -\mathbf{Z}_r^{-1} \begin{pmatrix} V_h (\mathbf{m}\cdot\nabla)(\mathbf{H}_r\mathbf{B}_w) \\ V_h \mathbf{m}\times(\mathbf{H}_r\mathbf{B}_w) \end{pmatrix} \tag{1}$$

where, $\mathbf{U}$ represents the linear velocity, while $\mathbf{\Omega}$ stands for the angular velocity of the MPMR. $\mathbf{Z}_r$ denotes the hydrodynamic resistance matrix of the entire MPMR. V_h signifies the volume of magnetic particles within the polymer matrix of the head, $\mathbf{H}_r$ represents the rotation matrix transforming between the reference frame of the MPMR and the laboratory reference frame, and $\mathbf{m}$ is the average magnetic moment of the MPMR. The $\mathbf{Z}_r$ is given by:

$$\mathbf{Z}_r = \int_0^L \begin{pmatrix} \mathbf{HCH}^T & -\mathbf{HCH}^T\mathbf{S}_t \\ \mathbf{S}_t\mathbf{HCH}^T & \mathbf{S}_t\mathbf{HCH}^T\mathbf{S}_t \end{pmatrix} dL \tag{2}$$

where, L denotes the length of the MPMR, $\mathbf{S}_t$ represents the skew symmetric matrix that satisfies the cross-product relationships. $\mathbf{C}$ and $\mathbf{H}$ are diagonal matrices that correspond to the coefficients of local resistive forces and the rotation matrix from local Frenet-Serret coordinate frames to the inertial frame, respectively, given by

$$\mathbf{C} = \begin{pmatrix} C_t & & \\ & C_n & \\ & & C_b \end{pmatrix} \text{ and } \mathbf{H} = [\mathbf{t} \quad \mathbf{n} \quad \mathbf{b}] \tag{3}$$

where, C_t, C_n and C_b represent the tangential resistance coefficient, the normal resistance coefficient and the binormal resistance coefficient, respectively, and are given by

$$C_t = -2\pi\mu / \left(\ln\left(\frac{L}{r_t}\right) + C_2 \right);\ C_n = C_b = -4\pi\mu / \left(\ln\left(\frac{L}{r_t}\right) + C_1 \right) \tag{4}$$

where, the value of C_2 was determined as C_1 -1, where the value of C_1 was contingent upon the axial variation in the radius of the cylinder. Specifically, for a cylinder with a uniform axial profile, the value of C_1 =ln2 -1/2. Conversely, in the case of a prolate spheroid, the value of C_1 =1/2. And the μ denotes the dynamic viscosity of the surrounding medium, and $r_t = \frac{1}{2}\sqrt{h^2 + b^2}$ represents the effective radius of the MPMR, which is defined with respect to a cylinder of identical radius and length. Additionally, $\mathbf{t}$, $\mathbf{n}$, and $\mathbf{b}$ refer to the local tangent, normal, and binormal vectors, respectively, that characterize the orientation and geometry of the MPMR, respectively:

$$\mathbf{t} = \frac{d\mathbf{p}/dx}{\|d\mathbf{p}/dx\|},\ \mathbf{n} = \frac{d\mathbf{t}/dx}{\|d\mathbf{t}/dx\|},\ \mathbf{b} = \mathbf{t}\times\mathbf{n} \tag{5}$$

where, $\mathbf{p} = [x \quad \varphi \quad z]^T$ denote the local deformation vector, while φ represents the deformation of the MPMR. The time-dependent Frenet-Serret frames ($\mathbf{t}$, $\mathbf{n}$, and $\mathbf{b}$), as referenced in equation (5), are derived by solving the structural deformation equation of the MPMR, specifically as follows:

$$\begin{aligned} EI\frac{\partial^4\varphi}{\partial x^4} = f_y + \left(J + \frac{mEI}{kAG}\right)\frac{\partial^4\varphi}{\partial t^2\partial x^2} + \frac{J}{kAG}\frac{\partial^2 f_y}{\partial t^2} \\ -\frac{EI}{kAG}\frac{\partial^2 f_y}{\partial x^2} - m\frac{\partial^2\varphi}{\partial t^2} - \frac{Jm}{kAG}\frac{\partial^4\varphi}{\partial t^4} \end{aligned} \tag{6}$$

where E represents the modulus of elasticity, and I represents the second moment of area of the MPMR. The lateral hydrodynamic force acting on the MPMR is denoted by f_y. Furthermore, J, m, and k represent the moment of inertia per unit length, mass, and shape correction coefficient,

respectively. The cross-sectional area and shear modulus of the MPMR are indicated by A and G, respectively. The local hydrodynamic forces acting on the MPMR are expressed mathematically as follows:

$$\begin{bmatrix} f_x & f_y & f_z \end{bmatrix}^T = \begin{bmatrix} \mathbf{HCH}^{\mathrm{T}} & -\mathbf{HCH}^{\mathrm{T}}\mathbf{S} \end{bmatrix} \begin{bmatrix} \mathbf{U} \\ \mathbf{\Omega} \end{bmatrix} \tag{7}$$

Among these, the thrust force f_{x} along the axial direction of the MPMR can be expressed as:

$$f_x = \int_0^L \frac{-v_x(C_t + \beta^2 C_n \sin(kx - \omega t)^2) - \beta v_y \sin(kx - \omega t)(C_n - C_t)}{\beta^2 \sin(kx - \omega t)^2 + 1} dx \tag{8}$$

Note S2. Magnetic Actuation and Experimental Setup

As shown in Fig. S2A, a magnetic field-driven system (working space: 110×110×110 mm) was developed to control a MPMR. The system consists of three-axis orthogonal circular Helmholtz coils, an industrial camera (AOSVI 3M280GS), a servo motor driver (Maxon ESCON 70/10), a host computer, and a DC power supply. It can generate a programmable magnetic field with a maximum intensity of 10 mT and a resolution of 0.1 mT.

According to the control signals $\mathbf{B}_{\mathrm{X}}$ and $\mathbf{B}_{\mathrm{Y}}$ of the X-field and Y-field shown in Fig. S2B, the overall actuation of the MPMR is achieved using an oscillating magnetic field in a uniform plane. The electromagnetic drive system provides a field intensity of up to 10 mT within the frequency range of 0-10 Hz. In the uniform field region at the center of the Helmholtz coils, the field gradient generated by the system does not affect the oscillation of the MPMR. Therefore, the propulsion of the MPMR is entirely due to lateral oscillations under dynamic torque. The main control parameters are adjusted by varying the current and oscillation frequency to regulate the magnetic field intensity.

The MPMR responds to changes in the direction of the driving field with different shapes. When the driving field oscillates, the MPMR deforms its body in a series of traveling waves, creating continuous left-right motions for propulsion in the fluid. To further study the impact of body structure changes during motion, a fluid-structure interaction simulation of the MPMR's undulatory movement was conducted using Abaqus finite element simulation software. The motion postures of the MPMR at different times and their simulated counterparts are compared in Fig. S2C. Under the cyclic drive of a 5 mT, 3 Hz oscillating field, the undulatory gait produces small displacements. The Reynolds number of the MPMR ranges from 0 to 30, depending on the vibration frequency, fluid viscosity, and MPMR size.

Note S3. Statistical Analysis of MPMRs Locomotion Performance with Morphology-Encoded Selective Actuation

GraphPad Prism 9 software (GraphPad Software) was utilized for statistical data analysis. Multiple comparisons were conducted using an ordinary one-way analysis of variance (ANOVA), followed by Tukey's multiple comparisons test. A p-value of less than 0.05 was considered statistically significant, with N.S. (p-value > 0.05), * (0.01 < p-value ≤ 0.05), ** (0.001 < p-value ≤ 0.01), *** (p-value ≤ 0.001), and **** (p-value ≤ 0.0001).

1. In vitro hydrodynamic environment

Tukey's post-hoc analysis revealed significant morphology-dependent velocity variations across all tested actuation frequencies (1-6 Hz). At 1 Hz, AP-14 exhibited a mean velocity deficit of 1.0 mm/s compared to AP-11 (Mean Diff. = -1.0, P < 0.0001), while AP-11 demonstrated superior performance over AP-41 (Mean Diff. = 0.8 mm/s, P = 0.0015). Frequency escalation amplified these disparities, with AP-14-AP-11 differentials expanding to -3.1 mm/s at 3 Hz (P = 0.0048) and AP-11-AP-41 differences reaching 5.0 mm/s at 6 Hz (P = 0.0081). The progressive enhancement of AP-14's relative performance at higher frequencies (e.g., +3.5 mm/s vs. AP-41 at 6 Hz, P = 0.0018) suggests morphology-specific hydrodynamic optimization principles. All inter-group

comparisons maintained statistical significance (adjusted $P < 0.05$) after multiple-test correction, confirming robust morphology-mediated propulsion control in unconfined fluidic environments.

2. In ex vivo intestinal tissue environment

Significant morphology-driven locomotion differentiation persisted in intestinal tissue models despite systemic velocity attenuation. AP-14 maintained consistent underperformance relative to AP-11 across frequencies (1 Hz: -0.9 mm/s, P=0.0061; 2 Hz: -1.3 mm/s, P=0.0063), though these differentials demonstrated reduced frequency sensitivity compared to in vitro conditions. Notably, AP-14 exhibited enhanced environmental adaptability, as evidenced by mitigated performance degradation against AP-41 at 2 Hz (Mean Diff. = -0.3 mm/s, P=0.0043 vs in vitro -0.53 mm/s). The preservation of statistically robust inter-morphology distinctions (all adjusted $P<0.01$) under complex biological constraints validates the clinical relevance of morphology-encoded control strategies. These findings collectively establish frequency-morphology interaction as a critical determinant of locomotion of MPMRs in both synthetic and biological media.

Note S4. Locomotion robustness under physiologically relevant viscosity and background flows

To assess the biomedical relevance of the proposed morphology-encoded locomotion and selective control strategy, we characterized the swimming performance of MPMRs in viscosity-modulated aqueous media. Methyl cellulose (MC) was used to prepare viscosity-modulated aqueous solutions. MC powder was dispersed in warm DI water (60–70°C) under vigorous stirring, followed by dilution to the desired concentration and degassing at 4°C overnight. Two MC solutions were prepared to emulate elevated viscosities representative of mucus-like environments: 0.3% (w/v) MC ($\mu \approx 4.3$ mPa·s) and 0.5% (w/v) MC ($\mu \approx 7.6$ mPa·s), in addition to water ($\mu \approx 0.93$ mPa·s) at 23 ± 1.5°C (**Fig. S8**). The viscosities of the aforementioned solutions were measured at room temperature in a rotational viscometer (NTV-79, Shanghai Ni Run Intelligent Technology Company, Shanghai, China).

Across all tested morphologies, increasing viscosity monotonically reduced the steady swimming velocity under identical magnetic actuation parameters, consistent with increased viscous drag and damping of the body wave propagation. In particular, the effective operating frequency range shifted toward lower frequencies as viscosity increased, indicating an earlier onset of step-out in more viscous media.

Importantly, morphology-dependent frequency responses were preserved in MC solutions, maintaining distinguishable velocity separations among different MPMRs under the same uniform field. This observation supports that the proposed selective actuation mechanism, which relies on relative velocity differentiation rather than binary on/off actuation, remains applicable under viscosity variations relevant to biomedical settings.

Furthermore, regarding the background flow speeds in biomedical applications, the physiological fluid flow generated by gastrointestinal peristalsis and secretion is typically slow, ranging from approximately 0.5 to 2 cm/min. In contrast, our MPMRs demonstrate a maximum active swimming speed of 5.6 mm/s (approximately 33.6 cm/min) under a 5 Hz magnetic field. This velocity is over 15 times higher than the typical background flow speed in the gastrointestinal tract, indicating that the MPMRs possess sufficient propulsion capability to overcome background flows and perform retrograde or precise navigation in these physiological environments.

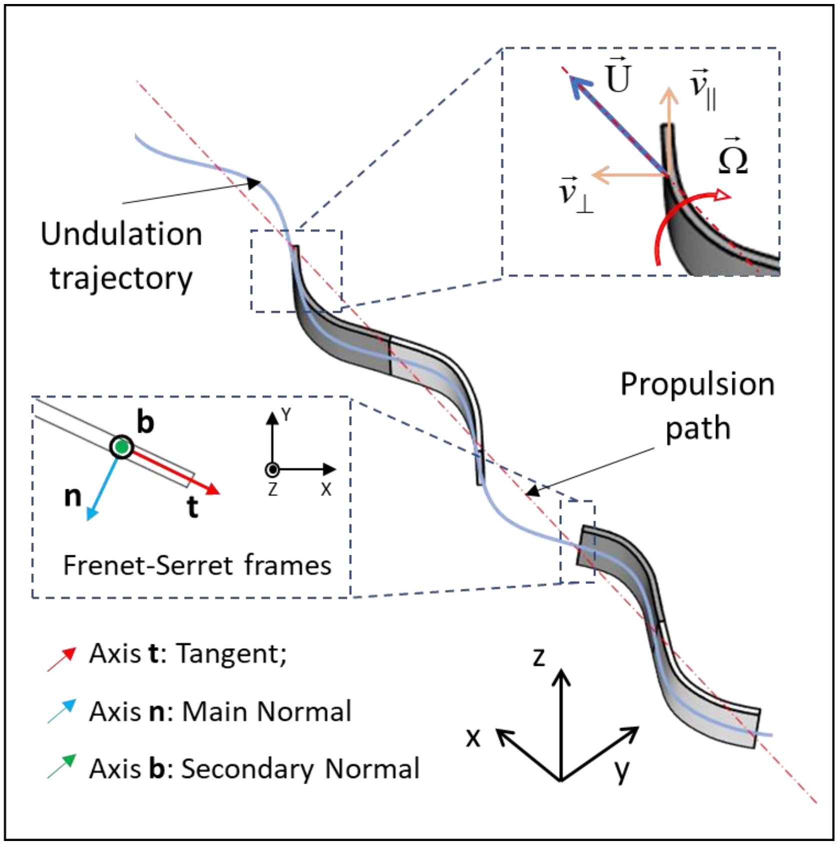

Fig. S1. Schematic diagram of the propulsion principle of the undulating motion

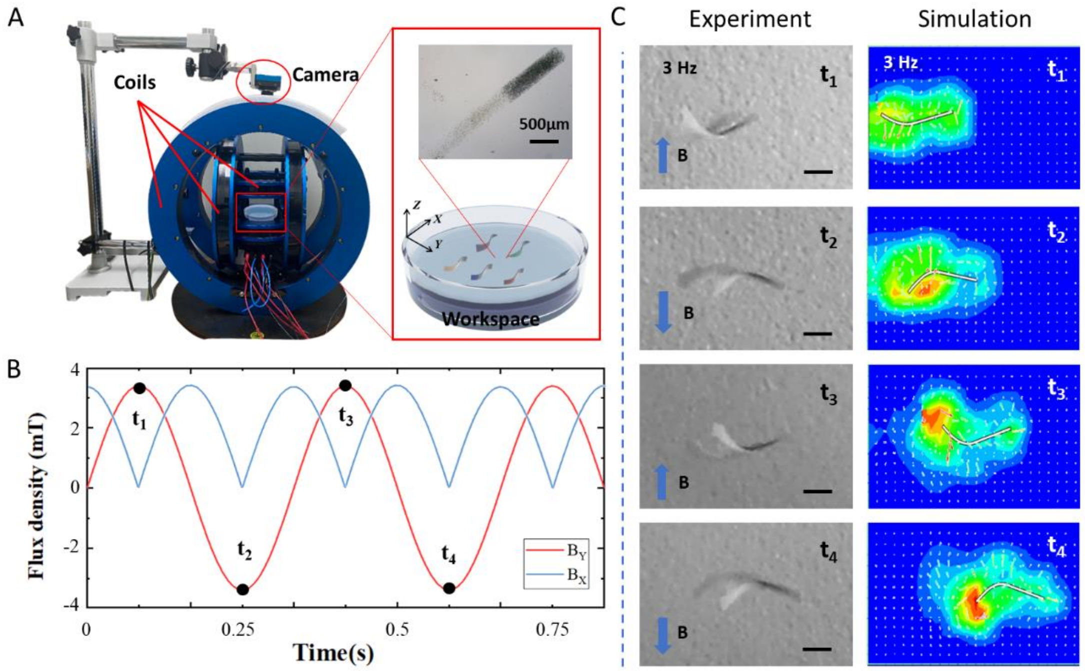

Fig. S2. Magnetic actuation and the experimental setups. (A) Physical image of the magnetic field drive system and the Milliobot. **(B)** A sample signal of the actuating uniform fields superposed by $\mathbf{B}_X$ and $\mathbf{B}_Y$. Oscillating magnetic field, 5 mT with 3 Hz is generated to control the MPMR. **(C)** A comparison of the experimental and simulated half-period near-cycling swimming kinematics of the MPMRs' undulatory motion is provided. Scale bar: 2 mm. The orientation of the magnetic flux density is indicated.

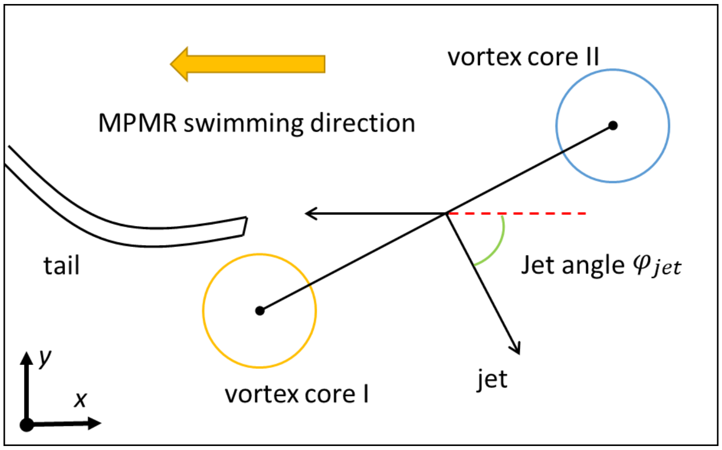

Fig. S3. Schematic illustration of the determination of jet angle (φ_{jet}) for the MPMR.

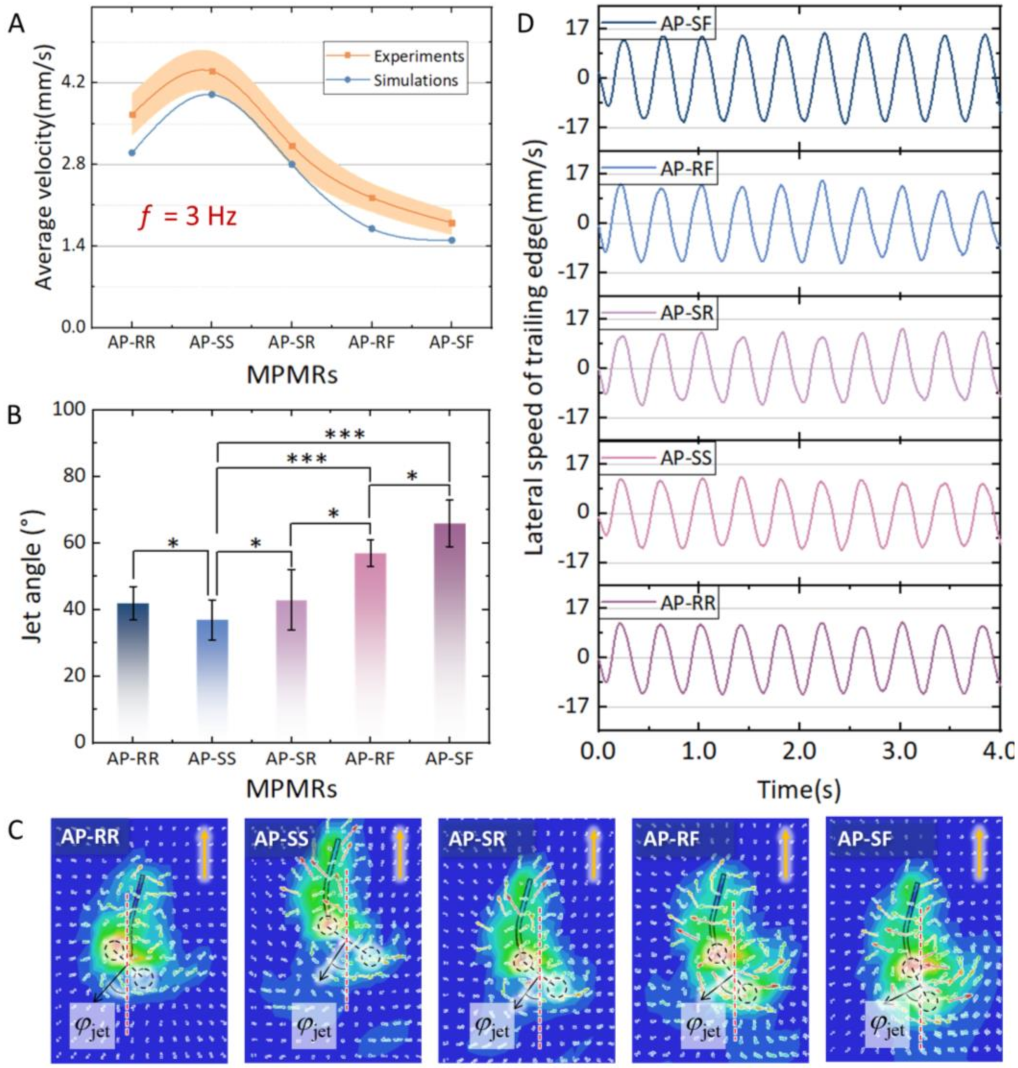

Fig. S4. Flow field and kinematic analysis on the undulatory motion of soft MPMRs with different body contours. (A) A comparison of the average velocity in experimental and simulation studies of undulatory motion in MPMRs. **(B)** Comparison of jet angles in the undulatory motion of MPMRs. The results reveal that the AP-SS demonstrates an 11.9% reduction in jet angle compared to the AP-RR. In contrast to the AP-SF the jet angle is diminished by an impressive 43.9%. (One-Way ANOVA) **(C)** Fluid-structure interaction phenomena in the flow field simulation of MPMRs undulatory motion (AP-RR, AP-SS, AP-SR, AP-RF, AP-SF). Results indicate that the AP-SS (streamline shape) exhibits a 11.9% reduction in jet angle $\boldsymbol{\varphi_{jet}}$ compared to the AP-RR (rectangle shape). Relative to the AP-SF (Fish tail shape), the jet angle $\boldsymbol{\varphi_{jet}}$ is reduced by 43.9%. **(D)** Lateral velocity variation curve at the tail edge of MPMRs. Compared to the AP-SF, the maximum lateral velocity at the posterior module edge of the AP-SS is elevated by 35.7%. Data are presented as mean ± SD (n = 5). Statistical analysis: *$p < 0.05$, **$p < 0.01$, ***$p < 0.001$, ****$p < 0.0001$.

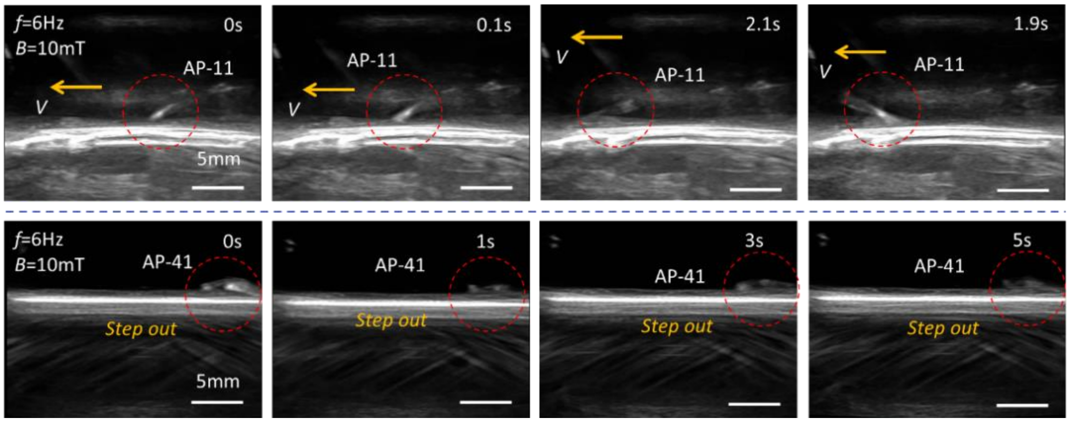

Fig. S5. Comparison of the motion performance of MPMRs in vitro fluidic environments and ex vivo porcine gastric tissue. (AP-11 and AP-41)

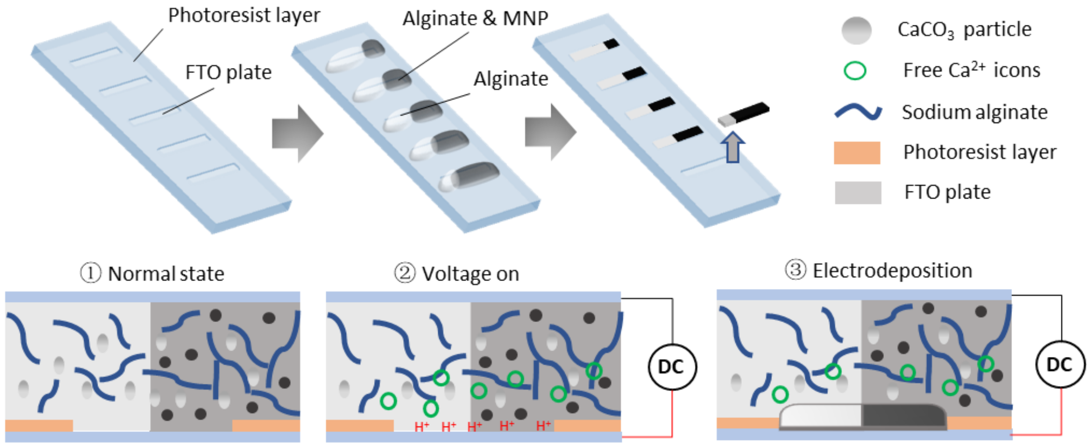

Fig. S6. Fabrication of the MPMRs. The fabrication of magnetic calcium alginate hydrogel microrobots (MPMRs) involves an electrodeposition-based process. A magnetic precursor solution, formulated with 1% (w/v) sodium alginate, 0.25-0.75% (w/v) calcium carbonate, and Fe_3O_4 nanoparticles, and a non-magnetic flexible solution were selectively dispensed onto patterned anodic substrates in adjustable ratios. The assembly was completed by overlaying a cathodic plate, after which a 3-5 V DC voltage was applied across the FTO layers for 5–10 seconds to induce localized electrodeposition. This triggered the formation of an anisotropic hydrogel structure through ionic crosslinking, with the magnetic anterior and flexible posterior modules determined by the spatial distribution of the precursor solutions. Subsequent magnetization of the substrate under a 1T directional magnetic field aligned the Fe_3O_4 nanoparticles, followed by gentle rinsing to release the MPMR. By modulating the precursor ratios and substrate patterning, the anterior-posterior proportions and macroscopic morphology of the microrobots could be programmably engineered to meet specific functional requirements.

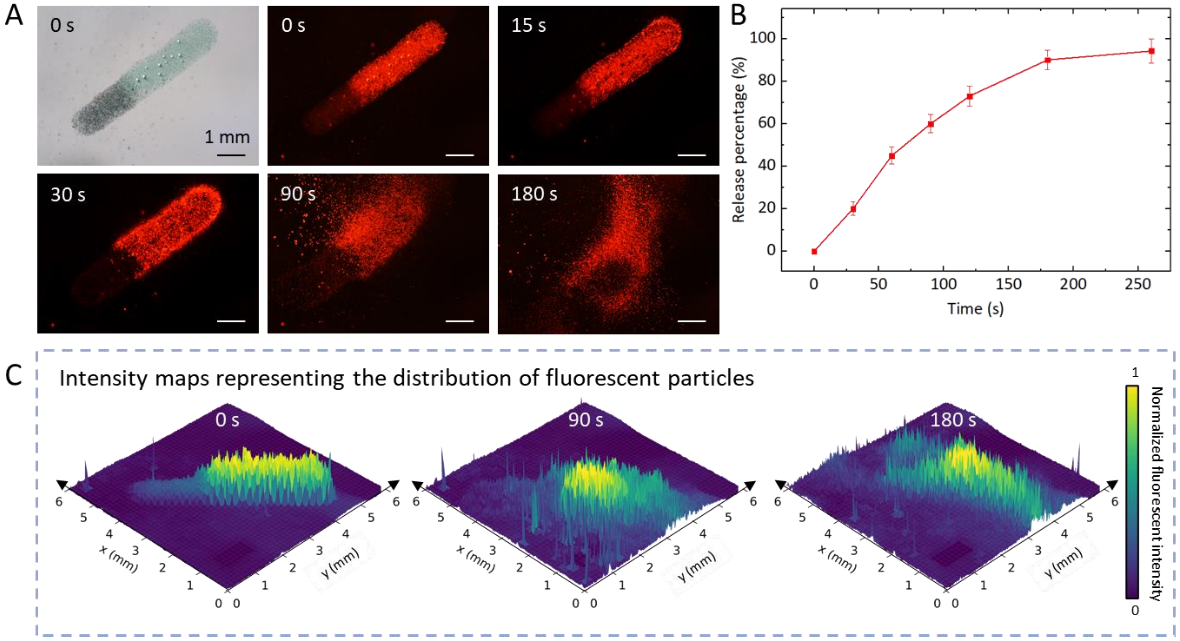

Fig. S7. Quantitative characterization of the dissolution-triggered payload release. (A) Bright-field and fluorescent images showing the distribution of fluorescent particles. Scale bar: 1 mm. **(B)** Release percentage–time profile of the effective payload from MPMRs under specified experimental conditions. Data are presented as mean ± SD (n = 5). **(C)** Intensity maps representing the distribution of fluorescent particles

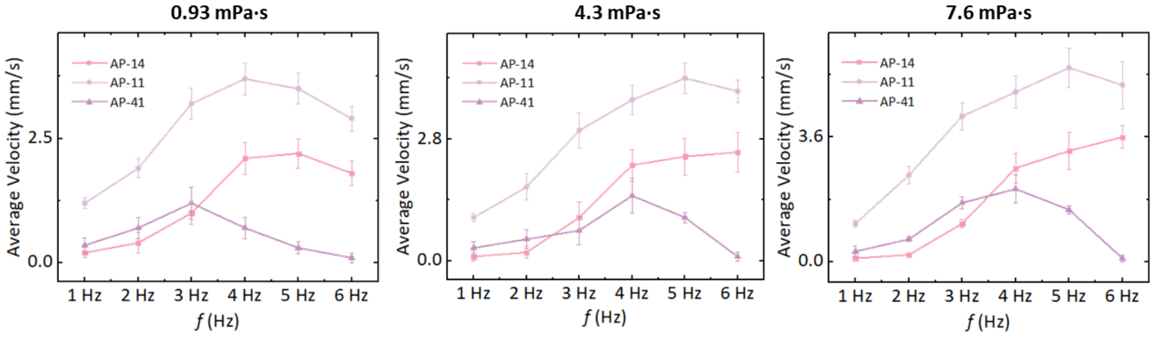

Fig. S8. Swimming performance of MPMRs in fluids with varying viscosities. Data are presented as mean ± SD (n = 5).

Table S1. Statistical analysis of MPMRs motion performance in vitro hydrodynamic environment

	Frequency	Tukey's multiple comparisons test	Mean Diff.	Adjusted P Value	Summary
Vitro Hydrodynamic Environment	1 Hz	AP-14 vs.AP-11	-1	<0.0001	****
	1 Hz	AP-14 vs.AP-41	-0.2	0.0367	*
	1 Hz	AP-11 vs.AP-41	0.8	0.0015	**
	2 Hz	AP-14 vs.AP-11	-2.38	0.0055	**
	2 Hz	AP-14 vs.AP-41	-0.53	0.0029	**
	2 Hz	AP-11 vs.AP-41	1.85	0.0063	**
	3 Hz	AP-14 vs.AP-11	-3.1	0.0048	**
	3 Hz	AP-14 vs.AP-41	-0.6	0.006	**
	3 Hz	AP-11 vs.AP-41	2.5	0.0045	**
	6 Hz	AP-14 vs.AP-11	-1.5	0.0321	*
	6 Hz	AP-14 vs.AP-41	3.5	0.0018	**
	6 Hz	AP-11 vs.AP-41	5	0.0081	**

Table S2. Statistical analysis of MPMRs motion performance in ex vivo intestinal tissue environment

	Frequency	Tukey's multiple comparisons test	Mean Diff.	Adjusted P Value	Summary
Ex vivo intestinal tissue Environment	1 Hz	AP-14 vs.AP-11	-0.9	0.0061	**
	1 Hz	AP-14 vs.AP-41	-0.2	0.0064	**
	1 Hz	AP-11 vs.AP-41	0.7	0.006	**
	2 Hz	AP-14 vs.AP-11	-1.3	0.0063	**
	2 Hz	AP-14 vs.AP-41	-0.3	0.0043	**
	2 Hz	AP-11 vs.AP-41	1	0.0065	**
	3 Hz	AP-14 vs.AP-11	-1.2	0.0049	**
	3 Hz	AP-14 vs.AP-41	0.2	0.006	**
	3 Hz	AP-11 vs.AP-41	1.4	0.0051	**
	6 Hz	AP-14 vs.AP-11	-1.4	0.0069	**
	6 Hz	AP-14 vs.AP-41	2.6	0.0055	**
	6 Hz	AP-11 vs.AP-41	4	0.006	**

Supplementary Movies

Movie S1. Kinematic comparisons between fish and MPMRs during one cycle of near-cyclic swimming.

Movie S2. Experimental study and midline kinematic postures of five MPMRs [AP-14, AP-23, AP-11, AP-32, AP-41] under a 5mT, 3Hz oscillating magnetic field.

Movie S3. Experimental study and midline kinematic postures of five MPMRs [AP-RR, AP-SS, AP-SR, AP-RF, AP-SF] under a 5mT, 3Hz oscillating magnetic field.

Movie S4. Demonstration of collective motion of multiple MPMRs.

Movie S5. Experiments on four selective actuation modes for multiple MPMRs [AP-14, AP-11, and AP-41].

Movie S6. Selective control of multiple MPMRs under a general experimental setup.

Movie S7. Collective operation of multiple MPMRs under a uniform field.

Movie S8. The MPMRs performed selective control movements in the ex vivo porcine stomach and successfully released fluorescent particles.

Movie S9. Ultrasound images of MPMRs moving in the ex vivo porcine small intestine.

Movie S10. Multi-robot selective control and multi-target delivery in an ex vivo rat intestine
